# Is It Time to Anticipate the Use of PARP Inhibition in Prostate Cancer Patients?

**DOI:** 10.3390/curroncol30090584

**Published:** 2023-08-30

**Authors:** Alessandro Sciarra, Valerio Santarelli, Lorenzo Santodirocco, Marco Frisenda, Stefano Salciccia, Paolo Casale, Flavio Forte, Gianna Mariotti, Martina Moriconi, Susanna Cattarino, Beatrice Sciarra, Giulio Bevilacqua, Alessandro Gentilucci

**Affiliations:** 1 Department Materno Infantile e Scienze Urologiche, University Sapienza, 00166 Rome, Italy; va.santarelli@uniroma1.it (V.S.); lorenzo.santodirocco@uniroma1.it (L.S.); marco.frisenda@uniroma1.it (M.F.); stefano.salciccia@uniroma1.it (S.S.); gianna.mariotti@gmail.com (G.M.); martina.moriconi@uniroma1.it (M.M.); susycat84@hotmail.it (S.C.); giulio.bevilacqua@uniroma1.it (G.B.); alegenti@yahoo.com (A.G.); 2 Urologic Division, Humanitas Hospital, Rozzano, 00100 Milan, Italy; paolo.casale@humanitas.it; 3 Urologic Division, Figliesancamillo Hospital, 00198 Rome, Italy; flavio.forte@figliesancamillo.it; 4 Department of Chemistry, University Sapienza, 00166 Rome, Italy; beatricesciarra@gmail.com

**Keywords:** prostate cancer, PARP inhibitors, DDR gene, CRPC

## Abstract

The increasing diffusion of genetic analysis regarding the pathogenetic variants (PVs) of genes involved in DNA Damage Repair (DDR) mechanisms and the development of Poly ADP ribose polymerase (PARP) inhibitors (PARPis) led to the first valid precision medicine option tailored toward metastatic prostate cancer (mPC). The concept of anticipation in the systemic treatment of mPC was initially adopted for androgen receptor signaling inhibitors (ARSIs) to describe the expansion of their indications, from the setting of the late-stage second-line treatment of metastatic castration-resistant prostate cancer (mCRPC) to first-line therapy in selected cases. There is already mounting evidence in favor of the anticipation of PARPis in the first line of mCRPC therapy, and further evidence in favor of mHSPC is emerging. Many studies have demonstrated the synergism between ARSIs and PARP inhibitors. Recent discoveries regarding the crosstalk between the androgen receptor (AR) and DNA repair mechanisms are disconnecting the use of PARPis from genetic analysis. The new message emerging is that the combination of PARPis with ARSIs may work independently of DDR mutational status. As a matter of fact, most of the recent trials analyzing the combination of PARPis with abiraterone or enzalutamide as a first-line therapy enrolled mCRPC patients irrespective of their mutational status. The PROPEL trial concluded that the advantage of the combination was independent of PV status, despite a higher advantage being reported in the BRCA1/2 mutated subgroup. The MAGNITUDE trial, however, showed a significant advantage only in the DDR mutated subgroup, and the DDR non-mutated cohort was closed for further enrollment. The combination of PARPis with ARSIs represents a significant strategy with a view to the anticipation and intensification of care in mPC. However, it should not nullify the advantages of precision medicine linked to the genetic analysis of DDR genes.

## 1. Introduction: The Concept of Anticipation in the Systemic Therapy of Prostate Cancer

For a long time, the natural history of metastatic prostate cancer (mPC) has been simply divided into two separate phases: 1. hormone-sensitive (HSPC) and 2. hormone-refractory (HRPC), subsequently better defined as castration-resistant (CRPC). These two phases were considered two distinct diseases with different therapeutic approaches, with the first subjected to different lines of manipulation of the classic androgen deprivation therapy (ADT), and the second to chemotherapy (CHT) with taxanes. The systemic treatment of mPC has undergone an epochal positive evolution in recent years, in particular with the development of new-generation hormone manipulations and androgen receptor signaling inhibitors (ARSIs). Most of these new therapies were initially indicated in the late stages of the disease (post CHT-mCRPC) [1,2] where options and patient survival were limited. However, several clinical trials have shown that ARSIs and CHT are equally effective in earlier stages, such as first-line mHSPC [3,4,5,6]. The intention of treating more patients for a longer time has led to the concept of **anticipation** in the systemic treatment of mPC, which brought several changes to therapeutic strategies, including the following: 1. the use of both docetaxel and ARSIs in mHSPC; 2. the stratification of mPC in classes such as high- and low-volume, high- and low-risk or de novo and progressive tumors; 3. competition in the use of these drugs between the mHSPC and mCRPC phases; and 4. the shift from a clear distinction between the HSPC and CRPC phases and their management to a merger of the two. At present, the management of mCRPC represents a second line of therapy, sharing several of the recommended drugs with mHSPC. The concept of the anticipation of therapy has merged with that of the intensification of care, leading to first-line therapeutic schemes with doublets or triplets of drugs in mHSPC. As a matter of fact, ADT alone no longer represents a therapeutic option in mHSPC, and the current recommendation is for an association with ARSIs, docetaxel or both [3,4,5,6]. In patients with high-volume/high-risk de novo mHSPC, the recent trials PEACE1 [7] and ARASENS [8] introduced the concept of triplets, with abiraterone or daralutamide in combination with docetaxel and ADT. 

**Figure 1:**
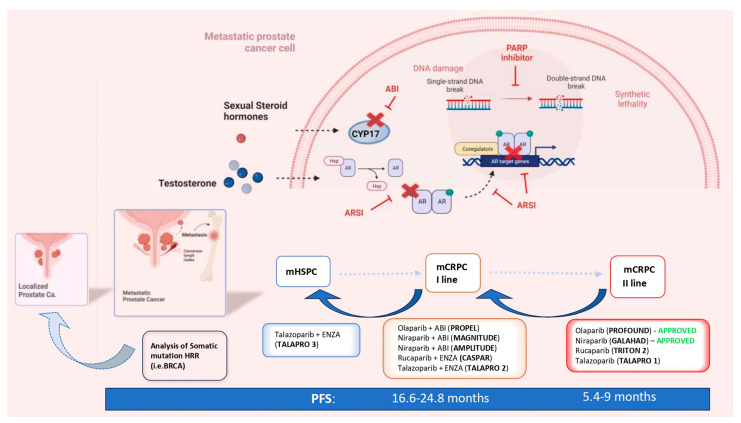
The concepts of the anticipation and intensification of care in the systemic therapy of metastatic PC and the ways in which PARP inhibitors and HRR genetic analysis are involved (mCRPC = metastatic castration-resistant prostate cancer; mHSPC = metastatic hormone-sensitive prostate cancer; nmCRPC = non-metastatic castration-resistant prostate cancer; PFS = progression-free survival; HR = hazard ratio; HRR = homologous recombinant repair; ABI = abiraterone; ENZA = enzalutamide; PARPi = Poly ADP ribose polymerase inhibitor).

The main criticalities associated with the concepts of anticipation and intensification of care are the development of early drug resistance and the limited choice of treatment for late mCRPC, with the need to repeat the same combination of drugs used as in first-line therapies for HSPC. The key turning point in this scenario, emphasized by the numerous therapeutic options available, is the identification of valid prognostic indicators of treatment response and the development of personalized therapies for each patient. The stratification of mPC on the basis of the volume, risk and development of the disease is not sufficient for precision medicine. The genetic analysis of the pathogenetic variants (PVs) of genes involved in DNA Damage Repair (DDR) mechanisms and the development of Poly ADP ribose polymerase (PARP) inhibitors (PARPis) led to the first valid precision medicine options tailored toward mPC. PARPis are following the same evolutionary path as that traced by ARSIs, expanding their indications starting from the late stage of second-line mCRPC treatment in a limited population and time frame. Inevitably, PARPis will also be absorbed by the concepts of the anticipation and intensification of care. There is already mounting evidence in favor of the anticipation of PARPis in the first line of mCRPC therapy, and further evidence in favor of mHSPC is emerging (Figure 1).

The purpose of this review is to analyze how the genetic analysis of DDR PVs and the clinical results of PARPis that are currently available can guarantee an anticipation process, always in compliance with precision medicine and tailored indications.

## 2. The Rationale and the Genetic Profiles That Sustain PARP Inhibitors in PC 

To address the underlying issue of genomic erosion, a complex network of DDR mechanisms has evolved [9,10]. Genetic abnormalities in DDR response systems are associated with life-threatening diseases such as immune deficiency, premature aging and cancer susceptibility. The following are the principal DNA repair pathways: single-strand break repair (SSR), mismatch repair (MMR), base excision repair (BER), homologous recombination repair (HRR), non-homologous end joining (NHEJ) and nucleotide excision repair (NER). With a single-strand break in DNA, the second intact strand represents a template for BER and NER repair. A double-strand break (DSB) activates the HRR, MMR and NHEJ mechanisms [11,12] 

PARP is a multifunctional protein that plays a critical role in SSR and BER mechanisms, primarily by recruiting DNA repair proteins to the sites of damage. So far, eighteen members of the PARP family have been found, among which PARP-1 is the most important [13]. PARP’s main enzymatic function is to add ADP-ribose to substrate proteins by cleaving NAD^+^ and releasing nicotinamide, thus activating a complex cascade, which ends in the recruitment of different proteins, such as DNA polymerase theta (POLQ), DNA ligase I and XRCC1, to the site of damage. Despite being well-known for its role in SSR and BER, recent evidence suggests that PARP can also affect DSB repair, mainly by controlling the expression of the key HR genes BRCA1 and RAD51 [14,15,16].

Defects in the DDR pathways can result in genomic instability and gene mutations, and, eventually, lead to the development of cancer. On the other hand, germline or acquired mutations in DDR also provide cancer-specific vulnerabilities that can be targeted by synthetic lethality-based therapies [17]. PARPis are the first successful example of a targeted therapy that uses synthetic lethality to kill cancerous cells with DNA-repairing deficiencies (e.g., BRCA1/2 mutation). These drugs act by competing with NAD^+^ for the catalytically active sites of PARP molecules, interfering with SSB repair by BER. The unrepaired SSB can be converted to DSB, and the primary mechanism in the repair of such lesions during the cell cycle is HRR. While HRR-proficient cells can repair the DSB originating from the SSB, thus ensuring genetic stability and cell survival, HRR-deficient cells are unable to do so, resulting in apoptosis and, ultimately, cell death [16,17,18]. Olaparib was the first drug to be developed in this group, initially approved in 2014 for patients with ovarian cancer with germline BRCA PVs and subsequently for those with breast, pancreatic and prostate cancer [19].

Germline HRR PVs increase the risk of developing PC by eight times at the age of 65 years and are linked to more aggressive PC, a higher risk of lymph-node invasion and distant metastasis at the time of diagnosis [20]. Moreover, germline BRCA 1/2 PVs are associated with a higher probability of failure in patients with localized disease undergoing active surveillance and a higher risk of recurrence in patients who have undergone curative treatment [21,22]. Several studies have analyzed the prevalence of germline and somatic mutations in HRR genes, both in metastatic and localized PC. A 2019 systematic review by Lang et al. [23] found a median prevalence of germline HRR PVs, and specifically of BRCA2 PVs, in patients with familiar PC rates of 29.3% (range, 7.3–91.67%) and 3.7% (range, 1.3–7.9%), respectively. In the same study, for unselected patients, the frequencies of somatic and germline mutations were, respectively, 3.9% and 1.5% for ATM, 1.1% and 0.6% for BRCA1, 4.9% and 1.1% for BRCA2, 1.3% and 0.5% for PALB2 and 1.5% and 0.5% for RAD51C. The overall prevalence of somatic DDR gene PVs ranged between 4.9 and 22%, while the rate of germline DDR PVs was 17.2–19%. 

Regarding metastatic PC, the incidence of germline PVs in HRR genes was found to be between 11% and 33% in a pan-cancer analysis of whole genomes [23]. Similarly, 8% of germline and 23% of somatic HRR PVs were found in 150 metastatic PCs by the International Stand Up to Cancer/Prostate Cancer Foundation team (SU2C-PCF) [24]. In this cohort, BRCA2 was the most prevalent mutation (13%) followed by ATM (7.3%), MSH2 (2%) and BRCA1. More specifically, in mCRPC, the incidence of somatic HRR PVs was 24% (BRCA 13%, ATM 7.3%, MSH2 2%, BRCA1 0.3%) in a study by Eeles et al. [25] and 28% in the Profound study, which analyzed the results of 2792 biopsies of mCRPC patients. 

Considering localized PC, the rates of PVs in HRR seem to be lower than those seen in mPC. Despite this, a 2019 study by Kim et al. [26] found an overall incidence of HRR pathway alteration in localized PC of 29.9%, higher than that suggested by a previous study by Marshall et al. [27] (11% in Gleason Grade Group 5 and 15.8% in cT3 patients).

The Profound study represents the biggest resource of tissue samples, with a total of 4858 centrally tested [28]. Next-Generation Sequencing (NGS) was performed mainly on primary tumor samples (83%). It is relevant to underline that of these primary samples, 96% were archival and 4% newly obtained. Samples from metastases were obtained in 17% of cases, and again, most were archival (60% archival and 33% newly obtained). This sample was found to be suitable for testing at similar percentages between the newly obtained and archival samples (63.9% vs. 56.9%) and between the metastatic and primary samples (63.9% vs. 56.2%). On the contrary, the NGS results significantly declined with increasing sample age. 

The somatic analysis of HRR mutations in PC should be used instead of germline analysis, when possible. A biopsy of metastatic lesions is the ideal method to identify the molecular changes that occur during disease progression. Despite this, biopsies of metastatic sites are challenging and not always feasible, and results from a single site may underestimate tumor heterogeneity. In the future, the analysis of free circulating DNA (cDNA) might overcome the issues associated with tissue biopsy, such as invasiveness, the need for anesthesia and pain. The first study to analyze cDNA in this field was the GHALAND study [29], where an analysis of treatment efficacy was performed on the basis of the amount of circulating tumor cells present from the eighth week of treatment. The BRCA cohort obtained better results when compared to the non-BRCA cohort, with a 24% CTC (circulating tumor cell) response. However, incongruences with the different commercial tests currently available for the analysis of cDNA are as high as 40%, with the risk of patients receiving inadequate or no treatment.

## 3. Clinical Trials and Actual Recommendations for PARP Inhibitors in Second-Line mCRPC 

The latest guidelines of the European Urological Association (EAU) recommend PARPis in pre-treated mCRPC patients with relevant DNA repair gene mutations.

So far, only two PARPis, olaparib and rucaparib, have been licensed by the FDA (the EMA have only approved olaparib), but other drugs of the same class are under evaluation (e.g., niraparib, talazoparib). The actual recommendations are supported by prospective trials analyzing a tailored therapy with PARPis for the treatment of mCRPC progressing after first-line treatment with ARSIs and taxanes.

In a second-line mCRPC setting, PARPis were analyzed as monotherapies in patients with HRR mutations. This type of analysis also determined the ranking of HRR PVs, with the BRCA2 mutation associated with the most significant benefits from all types of PARP therapy. 

The TOPARP-B trial [30], a multicenter, open-label, randomized phase II trial, was the first trial to investigate olaparib. All eligible patients had mCRPC and were required to have previously undergone at least one but no more than two taxane-based chemotherapy regimens (regardless of prior exposure to novel hormonal drugs). Genetic analysis by using NGS was performed, and all patients carried an allegedly pathogenic mutation or homozygous deletion in a DDR gene that could be related to susceptibility to PARPi. The 98 eligible patients with DDR gene aberrations were divided into two dose cohorts and randomly assigned to each one to receive 400 mg or 300 mg olaparib twice daily. Only the cohort receiving 400 mg twice daily met the predefined criteria for success, showing better percentages in all primary endpoints, which were as follows: radiological objective response, a 50% decrease in PSA from baseline and the conversion of circulating tumor cell count. Even though the results observed varied considerably for different HRR gene aberrations, the greatest antitumor activity was seen in the subgroup with BRCA1/2 mutations.

The Profound trial [31] can be considered the most relevant study in this field. A prospective, randomized, open-label, phase III trial was carried out at 206 sites in 20 countries and evaluated the safety and efficacy of olaparib in men with mCRPC who progressed while receiving a new hormonal agent (enzalutamide or abiraterone). 

Patients were divided into two cohorts depending on the qualifying gene alteration selected for their direct or indirect role in HRR; patients with at least one alteration in BRCA1, BRCA2 or ATM were assigned to cohort A, and patients with alterations in any of the other 12 genes were assigned to cohort B. In each cohort, patients were randomly assigned to receive olaparib (300 mg twice daily) or the physician’s choice of either enzalutamide (160 mg once daily) or abiraterone (1000 mg once daily) plus prednisone (control group).

Overall, 387 patients met all of the eligibility criteria, and thus, underwent randomization from April 2017 to November 2018. The primary endpoint was radiological progression-free survival (PFS), which was significantly longer in the olaparib arm (7.4 months vs. 3.6 months in the control group) (HR for progression or death, 0.34; 95% CI, 0.25–0.47; *p* < 0.001), especially in the cohort A patients (who had a 66% lower risk of disease progression or death).

In cohort A, as secondary endpoints, the confirmed objective response rate (ORR) was (olaparib vs. control group) 33% vs. 2%, a 50% reduction in PSA was seen in 43% vs. 8% and the median overall survival (OS) was 19.1 months vs. 14.7 months (HR 0.69, 95% CI 0.50–0.97, *p* = 0.0175).

The most common adverse events of any grade in the olaparib group were anemia (46% vs. 15%) followed by nausea and fatigue, and the overall incidence of grade ≥ 3 adverse events was higher in the olaparib group (51% vs. 38%).

The other FDA-approved PARPi, rucaparib, was evaluated in the TRITON-2 trial [32], an open-label, phase II study. A total of 115 patients with mCRPC who progressed after ARSIs or taxane and who had a pathogenetic variant in their HRR genes were enrolled. The ORRs (primary endpoint) were 43.5% (95% CI, 31.0% to 56.7%;) based on the radiology review and 50.8% (95% CI, 38.1% to 63.4%) considering the investigator assessment. No significant differences in ORRs were found between the germline and somatic BRCA variants or between the BRCA1 or BRCA2 variants, while the PSA response rate was higher in patients with a BRCA2 PV. PSA responses were greater in the *BRCA2* (59.8%) and biallelic patients (75.0%) compared to the *BRCA1* (15.4%) and mono-allelic patients (11.1%). The PSA response rate was 4.1% in the *ATM* group (49 patients), 6.7% in the *CDK12* cohort (15 patients) and 16.7% in the *CHEK2* group (12 patients). The ORRs were 10.5%, 0% and 11.1% in the *ATM* group, *CDK12* cohort and *CHEK2* group, respectively. The most frequent treatment-emergent adverse events (TEAEs) of any grade were asthenia (61.7%), nausea (52.2%) and anemia (43.5%).

The GALAHAD trial [29] is a multicenter, single-arm, phase II study that investigated the PARPi niraparib (300 mg once daily) as a second-line treatment in mCRPC cases. Patients were divided into two cohorts: one with germline/somatic PVs in BRCA1 or BRCA2 (BRCA cohort, *n* = 142) and one with other HRR PVs (non-BRCA cohort; *n* = 81). The median follow-up was 10 months. At final analysis, the ORR and the CRR were, respectively, 41% (95% CI 23.5–61.1%) and 63% (95% CI 47.6–76.8%) in the “*BRCA* group”, and 9% (95% CI 1.1–29.2%) and 17% (95% CI 6.6–33.7%) in the “non-BRCA group”. The median PFS and OS were 8.2 months (95% CI 5.2–11.1 months) and 12.6 months (95% CI 9.2–15.7 months) in the BRCA group, versus 5.3 months (95% CI 1.9–5.7 months) and 14.0 months (95% CI 5.3–20.1 months) in the non-*BRCA* cohort.

The most common TEAEs of any grade were nausea (58%), anemia (54%) and vomiting (38%); the most common grade ≥ 3 events were anemia, thrombocytopenia and neutropenia.

The last PARPi under investigation, talazoparib, was assessed in the TALAPRO-1 trial [33] (open-label, phase II). The inclusion criteria were the same as those of the GALAHAD trial, and eligible patients were given oral talazoparib (1 mg per day or 0.75 mg per day in patients with moderate renal impairment). Finally, 127 cases received at least one dose of the study drug (safety population) and 104 had HRR-deficient measurable disease (antitumor activity population). The primary endpoint was the confirmed ORR, whereas the secondary endpoints were the time to objective response, duration of objective response, proportion of patients with a decrease in PSA ≥ 50% from baseline, time to PSA progression, radiological PFS, OS and safety. After a median follow-up of 16.4 months, the ORR was 29.8% (95% CI; 21.2–39.6), 46% in cases with BRCA1/2 mutations with a radiological PFS of 11.2 months and 12% in cases with ATM mutations. Talazoparib produced a 50% PSA response in 66%, 7% and 6% of cases, and CTC conversion in 81%, 50% and 20%, in cases with BRCA 1/2, ATM and other mutations, respectively.

The most common grade ≥ 3 TEAEs in the overall population were anemia (31%), thrombocytopenia (9%) and neutropenia (8%).

## 4. The Prognostic Role of HRR PVs in Non-Metastatic and Metastatic HSPC as Indicators of Anticipation-Tailored Treatment 

It is widely accepted that BRCA2 gene mutations increase the risk of developing PC and are associated with an earlier onset, higher rates of lymph node invasion and distant metastasis at the time of diagnosis [34,35]. In this scenario, the IMPACT study evaluated the role of PSA screening in BRCA 1/2 PV carriers compared to non-carriers [36]. In total, 2932 patients were recruited; 919 were BRCA1 carriers, 709 BRCA1 non-carriers, 902 BRCA2 carriers and 497 BRCA2 non-carriers. After 3 years of follow up, the incidence of PC at biopsy was 5.2% in the BRCA2 group vs. 3.0% in the non-carrier cohort. In addition, BRCA2 gene PVs were associated with a higher incidence of intermediate-risk or high-risk disease (77% in BRCA2 carriers vs. 40% in non-carriers) and a younger age at diagnosis. The results of this study support the possibility of a tailored PV screening strategy for this more vulnerable population with a higher incidence of high-risk disease. 

Active surveillance (AS) is a well-established option for men diagnosed with favorable-risk and intermediate-risk PC. A study by Carter H.B. et al. [21] evaluated whether BRCA 1/2 and ATM PVs were associated with grade reclassification (GR) in patients undergoing AS, and thus, with the need for a more aggressive choice of treatment from diagnosis. Considering all 1211 participants, the rate of PV carriers was significantly higher in those reclassified both for the three-gene panel and for BRCA2 alone (3.8% and 2.1%, respectively) than those not reclassified (1.6% and 0.5%, respectively). However, the retrospective nature of this study and the absence of multiparametric magnetic resonance-targeted biopsies demand further evaluation of the role of AS in DDR PV carriers through prospective studies [37].

Castro E. et al. [22] evaluated the effect of BRCA PVs on metastatic relapse and cause-specific survival after radical treatment (surgery and radiotherapy) for localized disease. 1302 patients were enrolled, of which only 67 showed a germline BRCA alteration (18 for BRCA1 and 49 for BRCA2). This aspect limits the significance of a comparison between BRCA PV carrier and noncarrier populations. At 3, 5 and 10 years after primary treatment, metastatic-free survival was found in 97%, 94% and 84% of noncarriers vs. 90%, 72% and 50% of carriers. The percentage of cancer-specific survival (CSS) was significantly higher in the noncarriers (99%, 97% and 85%, respectively) than in the carrier group (96%, 76% and 61%, respectively; *p* < 0.001). Martinez Chanza M. et al. [38] retrospectively analyzed 380 men with localized and metastatic HSPC and showed a higher relapse risk in cases with BRCA alterations. Both authors suggest the use of a closer follow-up for patients with HRR PVs in a localized stage of PC, as well, and the need for randomized prospective clinical trials to confirm these data.

Different studies have analyzed the impacts of BRCA2 and other HRR PVs in metastatic PC. In a recent observational study, Antonarakis et al. [39] investigated the clinical impact of germline HRR PVs on the efficacy of first-line ARSIs among 172 mCRPC patients. Clinical/radiologic PFS, the primary endpoint, was longer both in patients with versus those without any germline DNA-repair PVs (median 13.3 vs. 10.3 months) and in those with versus those without BRCA/ATM PVs specifically (median 15.2 vs. 10.8 months). These results appear to be in conflict with a retrospective study of 319 patients with mCRPC by Annala M. et al. [40], which showed that HRR PV carriers had a significantly shorter PFS rate compared to non-carriers (3.3 vs. 6.2 months, *p* = 0.01). Despite this, another recent study [41], in accordance with Antonarakis et al. [39], suggested a better response to first-line abiraterone treatment in germline or somatic HRR PV carriers compared to non-carriers, supporting the idea of a “synthetic lethality” of treatment with more effective AR-targeted therapies in patients harboring germline HRR PVs.

Prorepair-B, a prospective multicenter cohort trial, was the first prospective study to evaluate the prognostic impact of BRCA1–2 and other HRR genes on CSS in mCRPC patients [42]. When taking into account all HRR mutations together, the authors found no significant difference in CSS between carriers and non-carriers (HRR PV carriers, 23.3 months vs. non-carriers, 33.2 months; *p* = 0.264). Surprisingly, there was statistical difference in CSS rates when only BRCA2 was considered (BRCA2 PV carriers, 17.4 months vs. non-carriers, 33.2 months; *p* = 0.027). Moreover, a subgroup analysis to determine whether the treatment sequence adopted had any effect on the carrier status’ impact on CSS was carried out. BRCA2 carriers were found to have worse outcomes compared to non-carriers when treated with the sequence docetaxel–ARSIs (median, 10.7 vs. 28.4 months; *p* < 0.001) but not when treated with the sequence ARSIs–docetaxel (median, 24.0 vs. 31.2, *p* = 0.901). Despite the need for validation in larger series, these results suggest that the prognostic impact of BRCA2 mutations may be influenced by the choice of first-line therapy.

## 5. PARP-AR Crosstalk and Current Clinical Trials with PARPis in Anticipated First-Line mCRPC 

### 5.1. Combination of PARPis and ARSIs Regardless of HRD Mutations

The understanding of the crosstalk mechanism between the AR and DNA repair is a topical object of research in mPC. The AR pathway regulates the transcription of the genes involved in DNA repair and androgen depletion interferes with HRR, rendering the tumor susceptible to PARPis [9,10]. On the other hand, the PARP system is involved in androgen-dependent transcription and PARP inhibition can impair it. Experiments showed that ARSIs are able to downregulate DDR gene expression in CRPC xenografts, ref. [43], resulting in decreased DNA repair in cells and increased double-stranded DNA breaks. This experimental evidence opens the possibility of new combination strategies with ARSIs and PARPi. It is interesting to highlight how, in this case, the development of this new combination therapy relates to the attempt to anticipate the use of PARPis and to expand their indication independently of HRR mutations in PC patients. The starting hypothesis is that the block produced by ARSIs on the AR pathway allows PARPis to act on PC cells independently from HRR mutations.

The major clinical trials underway analyzing this combination are anticipating its use as a first-line therapy in mCRPC. However, other trials are being defined to also extend the anticipation in an mHSPC setting. There are currently five major clinical trials evaluating the combination of PARPis and ARSIs in an anticipated phase of mCRPC. These trials, all phase 3 randomized placebo-controlled trials, are at different levels of evolution. The primary endpoint is radiologic PFS, and different approaches in terms of HRR mutations are used (Table 1).

### 5.2. PROPEL Trial: Abiraterone + Olaparib

The PROPEL trial (clinicaltrials.gov ID: NCT03732820) was recently concluded and published [44]. This double-blind, placebo-controlled, phase 3 trial enrolled 796 first-line mCRPC patients randomized to receive abiraterone plus olaparib or a placebo. Assignment in the two groups was stratified by distant metastasis type (bone, visceral, others) and by docetaxel treatment at the mHSPC stage. Enrollment was not based on HRR status; however, tissue and blood samples at baseline were collected and the results were also analyzed in terms of HRR PVs. The primary endpoint was radiological PFS, and OS was considered the secondary endpoint. Based on HRR PV analysis, 28.4% of patients were included in the HRR mutated subgroup. The radiological PFS was significantly longer in the abiraterone + olaparib than in the abiraterone + placebo arm (24.8 vs. 16.6 months; HR 0.66; 95% CI 0.54–0.81; *p* < 0.001). The advantage of the abiraterone + olaparib combination was observed across all subgroups (type of distant metastasis, previous docetaxel) irrespective of HRR status (HRR mutated subgroup: HR 0.50; 95% CI 0.34–0.73. HRR non-mutated subgroup: HR 0.76; 95% CI 0.60–0.97). Considering only BRCA PV, the advantage of the combination therapy over abiraterone alone was much higher in the BRCA mutated (HR 0.23; 95% CI 0.12–0.43) than in the non-mutated subgroup (HR 0.76; 95% CI 0.61–0.94). These data confirmed previous evidence from phase 2 studies on abiraterone + olaparib [45,46]. The most common side effects in the combination group were anemia (grade 3 in 15.1% in the abiraterone + olaparib and 3.3% in the abiraterone and placebo arm) and fatigue. The rate of cardiovascular events was similar between the two arms. The OS data were immature in the first analysis; however, at the ASCO GU 2023, a final analysis of OS for PROPEL patients [47] showed a 7-month advantage for the abiraterone + olaparib (42.1 months) when compared to the abiraterone + placebo (34.7 months) arm (HR 0.81; 95% CI 0.67–1.00; *p* = 0.0544). Also, for OS, the advantage of the combination therapy was observed in all subgroups irrespective of the HRR PV (HRR mutated subgroup: HR 0.66; 95% CI 0.45–0.95. HRR non-mutated subgroup: HR 0.89; 95% CI 0.70–1.14). In total, 374 patients had a subsequent therapy, most commonly chemotherapy, but for now, the data are too immature to calculate PFS2 (HR 0.76; 95% CI 0.59–0.99).

### 5.3. MAGNITUDE: Abiraterone + Niraparib

The MAGNITUDE trial (Clinicaltrials.gov ID: NCT03748641) was recently published [48]. This double-blind, placebo-controlled, phase 3 trial enrolled 423 first-line mCRPC patients randomized to receive abiraterone plus niraparib or a placebo. Assignment in the two groups was stratified by distant metastasis type (bone, visceral, others). Tissue and blood samples at baseline were collected and the results were also analyzed in terms of HRR PVs. The primary endpoint was radiological PFS, and OS was considered the secondary endpoint. In the BRCA1/2 mutated subgroup, median radiological PFS was significantly longer in the abiraterone + niraparib than in the abiraterone + placebo arm (16.6 vs. 10.9 months; HR 0.53; 95% CI 0.36–0.79; *p* = 0.001). Similarly, considering the HRR mutated subgroup, niraparib + abiraterone was associated with a longer radiological PFS (16.5 vs. 13.7 months; HR 0.73; 95% CI 0.56–0.96; *p* = 0.022). This result remained significant irrespective of the site of the metastases. In the HRR non-mutant cohort, no significant differences in terms of radiological PFS were found between the two treatment arms (HR 1.09; 95% CI 0.75–1.57; *p* = 0.66), and the cohort was closed to further enrollment. The results in terms of OS in the BRCA+ cohort are still immature (HR 0.88; 95% CI 0.58–1.34; *p* = 0.5505). The most common grade 3 adverse events were anemia (28.3% vs. 7.6%) and hypertension (14.6% vs. 12.3%) with abiraterone + niraparib versus abiraterone + placebo.

### 5.4. AMPLITUDE Trial: Abiraterone + Niraparib

The AMPLITUDE trial (Clinicaltrial.ov ID: NCT04497844) is an ongoing randomized placebo-controlled study on first-line mCRPC with HRR PVs (somatic or germline), comparing abiraterone + niraparib versus abiraterone + placebo [49]. The primary endpoint is radiological PFS, and OS is considered the secondary endpoint. The expected enrollment to be reached in November 2024 is 790 patients.

### 5.5. CASPAR Trial: Enzalutamide + Rucaparib

In the CASPAR trial (A031902; Clinicaltrials.gov ID: NCT04455750), an ongoing randomized phase 3 study, a total 984 first-line mCRPC patients will be randomized on a 1:1 basis to receive enzalutamide + rucaparib versus enzalutamide + placebo, irrespective of the HRR PV [50,51]. The co-primary endpoints are radiological PFS and OS. The OS analysis will be undertaken as a primary endpoint if the radiological PFS endpoint is met. The endpoints will be verified in subgroups with vs. without pathogenetic *BRCA1* and *BRCA2* mutations.

**Table 1:** Characteristics of 5 clinical trials analyzing the combination of ARSIs and PARP inhibitors in an anticipated first-line mCRPC setting (mCRPC = metastatic castration-resistant prostate cancer; mHSPC = metastatic hormone-sensitive prostate cancer; nmCRPC = non-metastatic castration-resistant prostate cancer; PFS = progression-free survival; HR = hazard ratio; HRR = homologous recombinant repair; PARPis = Poly ADP ribose polymerase inhibitors).

Study	Design	Treatments	Patient Selection	Primary Endpoint	Main Results
PROPEL [44]	Phase 3, randomized, placebo-controlled, double-blinded	Abiraterone + olaparib (360) Abiraterone + placebo (360)	First-line mCRPC Prior docetaxel allowed for mHSPC Prior ARSIs not allowed HRR mutation not required (but analyzed as subgroup)	Radiographic PFS	Radiological PFS 24.8 vs. 16.6 months; HR 0.66; 95% CI 0.54–0.81; *p* < 0.001, irrespective of HRR status (HRR mutated subgroup: HR 0.50; 95% CI 0.34–0.73. HRR non-mutated subgroup: HR 0.76; 95% CI 0.60–0.97). Overall survival 42.1 vs. 34.7 months; HR 0.81; 95% CI 0.67–1.00; *p* = 0.0544, irrespective of HRR mutation (HRR mutated subgroup: HR 0.66; 95% CI 0.45–0.95. HRR non-mutated subgroup: HR 0.89; 95% CI 0.70–1.14)
CASPAR [50]	Phase 3, randomized, placebo-controlled, double-blinded	Enzalutamide + rucaparib (496) Enzalutamide + placebo (496)	First-line mCRPC Prior docetaxel allowed for mHSPC Prior ARSIs allowed for mHSPC and nmCRPC HRR mutation not required (but analyzed as subgroups)	Radiographic PFS and overall survival	Ongoing
MAGNITUDE [48]	Phase 3, randomized, placebo-controlled, double-blinded	HRR-mutant cohort Abiraterone + niraparib (200) Abiraterone + placebo (200)	First-line mCRPC Prior ARSIs not allowed Prior docetaxel allowed for mHSPC	Radiographic PFS	In HRR mutant: PFS 16.5 vs. 13.7 months; HR 0.73; 95% CI 0.56–0.96; *p* = 0.022. In HRR non-mutant: HR 1.09; 95% CI 0.75–1.57; *p* = 0.66, and the cohort was closed to further enrollment.
		HRR non-mutated cohort Abiraterone + niraparib (300) Abiraterone + placebo (300)	First-line mCRPC Prior ARSIs not allowed Prior docetaxel allowed for mHSPC	Radiographic PFS	
TALAPRO-2 [52]	Phase 3, randomized, placebo-controlled, double-blinded	Enzalutamide + talazoparib (402) Enzalutamide + placebo (403)	First-line mCRPC Prior docetaxel allowed for mHSPC Prior abiraterone allowed (no novel AR inhibitors) for mHSPC and nmCRPC HRR mutation not required but analyzed as subgroups	Radiographic PFS	Median rPFS was not reached for talazoparib plus enzalutamide, and 21.9 months (95% CI: 16.6–25.1) was reached for placebo plus enzalutamide (hazard ratio 0.63; 95% CI 0.51–0.78; *p* < 0·0001)
AMPLITUDE [49]	Phase 3, randomized, placebo-controlled, double-blinded	Abiraterone + niraparib (395) Abiraterone + placebo (395)	First-line mCRPC with HRR pathogenetic variants Prior ARSIs not allowed		Ongoing

### 5.6. TALAPRO-2: Enzalutamide + Talazoparib

The TALAPRO-2 trial (Clinicaltrials.gov ID: NCT03395197) is a phase 3 placebo-controlled study on first-line mCRPC patients (with and without HRR alterations) randomized to receive enzalutamide + talazoparib vs. enzalutamide + placebo [52]. The enrollment goal is 805 patients in the following two cohorts: an all-comers cohort where patients are not required to have HRR alterations and an HRR PV cohort. The primary endpoint in both cohorts is radiological PFS, and OS is considered the secondary endpoint. A median follow-up of 24.9 months for the talazoparib group and 24.6 months for the placebo group was reached. Median rPFS was not reached (95% CI 27.5 months—not reached) for the talazoparib-plus-enzalutamide group and it was 21.9 months (95% CI 16.6–25.1) for the placebo-plus-enzalutamide group (hazard ratio 0.63; 95% CI 0.51–0.78; *p* < 0·0001). In the talazoparib group, the most common adverse events were anemia, neutropenia and fatigue (anemia 46%).

## 6. Conclusions

Therapeutic strategies for the treatment of metastatic PC have undergone radical changes in recent years. These changes can be summarized in the search for the anticipation of a cure, intensification through the combination with therapeutic doublets or triplets and the pursuit of a personalized medicine with precision. PARPis are also affected by these strategies. Evidence in favor of the anticipation of their indication from the late phase of mCRPC to first-line treatment of mCRPC is already available, and further evidence regarding their use in mHSPC will soon be published. In parallel, the concept of the intensification of care has led to the combination of PARPis with abiraterone and enzalutamide. Unlike previous strategies, PARPis represent the first evidence of a precision medicine in PC linked to the presence of PVs in HRR genes. The link between PARP and genetic analysis enhances therapeutic indication and paves the way for characterization of the genetic risk, anticipated from the metastatic to the non-metastatic phase of PC.

In contrast with this strategy, the new message emerging is that the combination of PARPis with ARSIs may work independently of genetic analysis and may be indicated as a first-line therapy regardless of the HRR mutations. Most of the recent trials analyzing PARPis plus abiraterone or enzalutamide enrolled first-line mCRPC patients irrespective of their HRR mutational status. In both the PROPEL and MAGNITUDE trials, the combination therapy showed a significant advantage in terms of radiological PFS when compared to abiraterone alone. Despite the highest advantage reported in the BRCA 1/2 mutated subgroup, the PROPEL trial concluded that the benefit of the combination therapy was independent of HRR status. The MAGNITUDE trial, however, showed a significant advantage only in the DDR mutated subgroup, and the DDR non-mutated cohort was closed for further enrollment. Moreover, radiological PFS is a relevant endpoint but we are waiting for mature data in terms of overall survival. It is not unrealistic to suggest that, considering this major endpoint, the influence of HRR mutations could become stronger. The combination of PARPis with ARSIs represents a significant strategy in the concept of the anticipation and intensification of care in metastatic PC. However, it should not nullify the advantages of precision medicine linked to the genetic analysis of DDR PV.

## Data Availability

Not applicable.
